# An Investigative Study on the Effect of Pre-Coating Polymer Solutions on the Fabrication of Low Cost Anti-Adhesive Release Paper

**DOI:** 10.3390/nano10081436

**Published:** 2020-07-23

**Authors:** Semen Vasilev, Andrey Vodyashkin, Daria Vasileva, Pavel Zelenovskiy, Dmitry Chezganov, Vladimir Yuzhakov, Vladimir Shur, Emmet O’Reilly, Alexandr Vinogradov

**Affiliations:** 1 Department of Chemical Science, Bernal Institute, University of Limerick, V94 T9PX Limerick, Ireland; semen.vasilev@ul.ie (S.V.); emmet.oreilly@ul.ie (E.O.); 2 School of Natural Sciences and Mathematics, Ural Federal University, 620000 Ekaterinburg, Russia; daria.vasileva@ul.ie (D.V.); zelenovskiy@urfu.ru (P.Z.); chezganov.dmitry@urfu.ru (D.C); vladimir.juzhakov@urfu.ru (V.Y.); vladimir.shur@urfu.ru (V.S.); 3 Peoples’ Friendship University of Russia (RUDN University), 6 Miklukho-Maklaya St., 117198 Moscow, Russia; av.andrey2013@yandex.ru; 4 ChemBio Cluster, ITMO University, 9 Lomonosova str., 191002 St. Petersburg, Russia; 5 Physical Department, University of Limerick, V94 T9PX Limerick, Ireland

**Keywords:** release paper, polymer coating, atomic force microscopy, Raman spectroscopy, PEVA

## Abstract

This work describes a novel approach to produce high quality release paper at lower cost than traditional methods. The anti-adhesive properties of release paper require the use of expensive machine glazed kraft or “Glassine” paper as the paper base. A series of polymer coatings including polyvinyl alcohol, carboxymethyl cellulose, polyethylene vinyl acetate, and polystyrene were chemically synthesized and coated onto a low cost pulp paper base. Surface roughness (Sa) and smoothness coefficients (k) were determined by atomic force microscopy (AFM), and the interactions between the polymer coating and base paper were investigated by Raman spectroscopy. Studies show the use of polyethylene vinyl acetate (PEVA) as a pre-coating layer on low cost pulp paper exhibits similar anti-adhesive properties as higher cost paper bases. In low margin markets such as the production of release paper, decreases in cost are critical to industry survival.

## 1. Introduction

Release paper (RP) is an integral part of everyday goods such as stickers, packaging, and hygiene materials. In addition it acts as protection for adhesive surfaces by preventing them from prematurely adhering [1,2]. Coating one or both sides of a base paper with a release agent provides a release effect against any type of sticky material [3]. Release agents typically consist of a variety of epoxysilicone blends with different additives. Silicone based coatings are hydrophobic, have good release properties due to a decrease in adhesion by 90–100%, and also assist in making the product waterproof [4]. Epoxysilicone blends, however, are expensive, and as such manufacturers will often try to limit the quantity required for release paper production. The uniformity, roughness, and porosity of the paper surface directly influences the quantity of epoxysilicone required for paper coating [5]. The surface morphology of base paper can vary significantly depending on the composition of raw materials and the paper manufacturing process. However, the influence of surface roughness, surface topography, and the presence of functional groups can be mitigated against by treating the paper with an additional pre-coating blend [6]. The pre-coating blend decreases overall surface roughness by filling the individual pores and cavities on the surface of the base paper, thus reducing epoxysilicone requirements. [7]. In addition, if the pre-coating layer has a strong adhesion for both epoxysilicone blends, the amount of the epoxysilicone required can also be reduced [8].

Machine glazed kraft paper (MG kraft), “Glassine” paper, or clay coated paper are the most popular materials used as RP bases [9]. Many release paper coatings exhibit reduced adhesion to these types of paper thereby complicating the manufacturing process. In addition, the adhesive side of MG paper is rough and porous thereby requiring large quantities of release agents [6,10].

To reduce the amount of coating material required, RP bases should ideally be 1) of high strength and capable of withstanding the printing process, 2) contain little defects or contaminants, 3) possess uniform thickness, and 4) have surface a roughness of approximately 100 nm and a contact angle of at least 95 degrees [11,12]. 

Polyvinyl alcohol (PVOH) has previously been used as a pre-coating blend to reduce surface roughness [11]; however, it was found that the subsequent silicone coating had poor penetration into the paper volume resulting in reduced adhesion to the paper. Attempts to remedy this issue have proven both expensive and hazardous due to the requirement to use environmentally harmful reagents [12,13,14,15].

An alternative approach to reduce cost in release paper manufacture is to use cheaper quality paper as an alternative to MG kraft paper. This approach requires the pre-coater blend to create a very smooth layer on the paper surface similar to expensive kraft paper. In addition, the pre-coating layer must display good adhesion to the paper base. 

This work describes the development of a low cost and green approach for producing release paper. By combining a unique pre-coating blend with low cost OPD label paper, a product with similar properties to more expensive MG kraft paper can be produced. OPD paper is not commonly used for release products, but it is affordable and widespread as it is used as a paper for offset printing.

Four different polymers were evaluated as active components for pre-coating blends with OPD paper. PVOH is a well-known component, which is often used for various applications in the paper industry, as it smooths out paper irregularities very well [16]. Carboxymethyl cellulose (CC) is a widely used material for surface modification of paper, usually used with various additives [17]. Polystyrene (PS) coatings had previously showed a good contact angle and release properties during preliminary experiments. Polyethylene vinyl acetate (PEVA) was chosen as a test coating, because the molecule has both a hydrophilic and hydrophobic group. Moreover, PEVA and EVA copolymers are the most commonly used as industrial adhesion agents and glues [18,19]. We provide an analysis of the surface properties of paper samples treated with different pre-coating blends and the functional properties of these release paper samples post siliconization. A comparative description of various pre-coating blends is shown, and the effectiveness of roughness reduction and paper–silicone adhesion are evaluated.

## 2. Materials and Methods 

### 2.1. Materials

Label paper OPD (Arkhangelsk Pulp & Paper Mill, Novodvinsk, Arkhangelsk region, Russia) was used as a base for sample preparation. Release agent was provided by the industry partner and consisted of an epoxysilicone blend with photocatalyst (Momentive SilForce UV9390C, Waterford, NY, USA) in the solution of ethyl acetate (99.8%, 270989). Styrene (99%, W323306), potassium peroxodisulfate (KPS) (99%, 60489), sodium chloride (99.5%, S9888), and CC (C4888, medium viscosity) were used for pre-coating layer active component preparation. All these reagents were purchased from Sigma-Aldrich Co (St. Louis, MO, USA) and were used as received without further purification. Deionized water was used in all the experiments. PEVA (55% water emulsion, Dairen Chemical Corporation, Taiwan) and PVOH (purity > 93.5%, dynamic viscosity 13–17 mPas, (JSC LenReactiv’s, Saint-Petersburg, Russia)) were provided by industrial partner Arkhangelsk Pulp & Paper Mill.

### 2.2. Methods 

#### 2.2.1. Synthesis of Pre-coating Solutions

1. PS was synthesized according to the procedure described by Keller et al. [20]. Monodispersed PS was synthesized by emulsifier-free emulsion polymerization of styrene using a modified procedure by Fang et al. [21]. In a typical synthesis, deionized water (150 mL) was placed in a 250 mL flask, followed by styrene (7.7 g), KPS (0.06 g), and NaCl. The synthesis was conducted at constant stirring (800 rpm) by magnetic stirrer, and the temperature of water bath was maintained at 80 °C. Post synthesis, the solution was evaporated under vacuum leaving behind the polymer particles. The solution of Styrene dispersion was then diluted by deionized water to final concentrations ranging from 4 to 0.1% (with step 0.5%). Nine concentrations were produced in total.
2. PEVA emulsion was diluted with deionized water to final concentrations ranging from 9.5 to 0.5% of PEVA. There were 11 concentrations in total.
3. The CC powder was placed in a beaker, and water was added to bring the final concentrations to between 0.25 and 2% (with steps of 0.25%). The solution was heated at 75 °C for 45 min with overhead stirring at 250 rpm.
4. PVOH was diluted by deionized water to concentrations ranging from 9.5 to 2% (with steps of 0.5%). Solutions were stirred by magnetic stirrer while heating to 90 °C for 90 min. Eleven concentrations were produced in total.

#### 2.2.2. Paper Coating

Label paper was used as a base for paper samples. Paper samples were covered with pre-coating blends using a commercial rotary printing press (experiments were done at the industrial partner equipment in the factory) (Supplementary Materials Figure S1) with a paper movement speed of 100 m/min and a drying temperature of 100 °C for 8 h. Once dried, the paper samples were covered by epoxysilicone blend containing the photocatalyst and polymerized in the presence of UVA light for 1 min.

Samples without the epoxysilicone layer were used for AFM, SEM, and Raman spectroscopy measurements to determine the effect of the coatings on the surface morphology of the paper. Samples with an epoxysilicone layer were used for adhesive tape peel and ink drops tests to assess the effect of the individual pre-coating layers in release paper applications. 

#### 2.2.3. Atomic Force Microscopy

Atomic force microscopes Solver Next (NT-MDT SI, Zelenograd, Russia) and Asylum MFP-3D (Asylum Research, Goleta, CA, USA) were used to study surface morphology and conduct roughness measurements. Images were obtained using semi-contact AFM mode with NSG01 probes, resonance frequency of 120–150 kHz, a radius about 10 nm, and a stiffness of 4–6 N/m. Image analysis was carried out using software by Gwyddion. Surface roughness values (Sa) and smoothness coefficients (k) were calculated as an average of a minimum 5 measurements obtained from different positions on the sample surface.

#### 2.2.4. Scanning Electron Microscopy

Scanning electron microscopy (SEM) imaging of paper samples was performed by MERLIN (Carl Zeiss, Oberkochen, Germany) in secondary electron detection mode. A built-in local charge compensation system by means of nitrogen gas blowing was used to avoid charging of the sample surface. An accelerating voltage of 3–5 kV and electron beam current of 150 pA were utilized.

#### 2.2.5. Raman spectroscopy

Polarized Raman spectra were measured using a confocal Raman microscope Alpha 300AR (WiTec GmbH, Ulm, Germany) equipped with a solid-state laser with wavelength 488 nm and maximum power of 27 mW. The incident laser beam was focused at the sample surface using a 100x objective with NA = 0.75. Diffraction grating with 600 grids per mm was used for the decomposition of the scattered light. Spectral resolution of the grating was 3.19 cm^–1^ for the laser wavelength used. Spectra were detected by CCD camera with 1600 × 200 pixels electrothermally cooled down to –60°C. Multimode optical fiber with a diameter of 50 μm was used as a confocal pinhole.

#### 2.2.6. Contact Angle Measurements

A shape drop analyzer DSA25 (Kruss, Hamburg, Germany) was used for contact angle measurements, and all measurements were performed at 20 °C. Each measurement represented the average contact angle for 5 samples prepared with the same active component concentration.

#### 2.2.7. Adhesive Tape Peel Test

The standard adhesive tape peel test EN 1939 [22] was used to check the quality of each of the release papers produced. Pieces of 3M “Scotch” adhesive tape were glued to the surface of PR samples containing each of the pre-coating layers. After 24 h of exposure, a 180° peel test was performed. The mass of samples was weighed before and after peeling off. The RP quality was evaluated by the mass difference (Supplementary Materials Table S1).

#### 2.2.8. Ink Drop Test

The ink drop test was used to determine the quality of pre coated RP. Water based black offset print ink with a volume of 0.2 mL was placed on the RP surface; after one minute, the drop was removed by filter paper. The quality of RP was evaluated by the amount of ink left on the RP surface.

## 3. Results and Discussion

### 3.1. Optimal Pre-coating Blends Concentrations

The pre-coating layers consisted of water and one of the active components PS, PEVA, PVOH, and CC. To choose the optimal active component concentrations for each blend, the paper was covered with pre-coating blends with different concentrations of active component using a commercial rotary printing press for offset printing. Then, coated paper was subjected to siliconization. 

**Figure 1:**
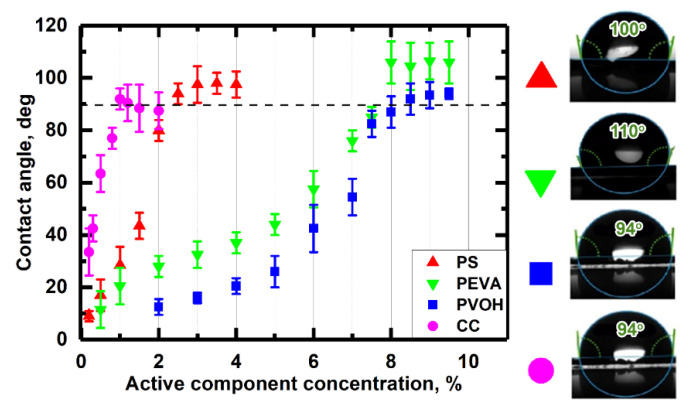
The contact angle dependence on the mass fraction of applied active component in the pre-coating layer blend. Pre-coating blend concentrations were as follows: 0.2 to 3% for PS, 0.2 to 9% for PEVA emulsion, 0.2 to 9.5% for PVOH solution, and 0.2 to 2% for CC solution.

Figure 1 shows the contact angles of the siliconized paper samples post drying. The optimal concentrations of pre-coating layer blends were determined on the basis of the smallest concentration of active component needed to achieve the 90° contact angle required for anti-adhesive properties. As can be seen from the Figure 1, the contact angle for each pre-coating blend reached constant value at a certain active component concentration. Further increase of the active component concentration did not lead to improvement of release paper anti-adhesive properties. Therefore, the optimal active component concentrations for each pre-coating blend were identified as those that allowed maximum contact angle with the minimum amount of active component (Table 1). All further experiments were performed using samples prepared with optimal active component concentration pre-coating blends.

### 3.2. Surface Study

**Figure 2:**
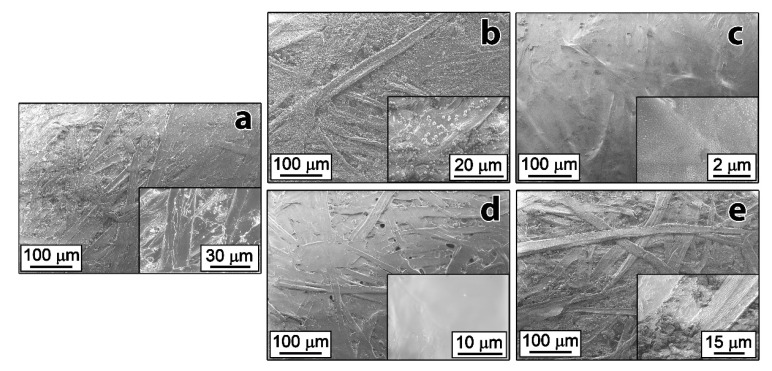
SEM images of paper surface (**a**) before and (**b**–**e**) after treatment with pre-coating blends: (**b**) 3% PS suspension, (**c**) 8% PEVA emulsion, (**d**) 8% PVOH solution, and (**e**) 1% CC solution.

SEM was used to examine the surface morphology of the paper bases after addition of the pre-coating layers. Figure 2a shows SEM images of uncoated paper highlighting the surface roughness and cellulose fibers present on the surface. Figure 2b shows the paper coated with PS particles as the pre-coating layer. The PS spheres were visible on the surface; however, coverage was limited, and significant surface roughness was still observed. Although the PS spheres covered the paper surface, they did not smoothen it, adversely affecting the quality of the coating. The high contact angle created by PS spheres lying separately could be attributed to the Lotus effect [23]. Figure 2c shows the paper surface fully covered by the PEVA polymer particles. The PEVA pre-coating layer produced a significantly smoother surface than polystyrene. Paper coated with PVOH (Figure 2d) also displayed visible smoothness. Figure 2e does not show any visible changes of the paper surface after applying the CC pre-coating layer and as such it was determined that CC did not meet the requirements to act as a successful pre-coating blend. Increasing the concentration of the CC blend beyond 2% resulted in a solution that was highly viscous and unsuitable for use in an industrial setting. 

**Figure 3:**
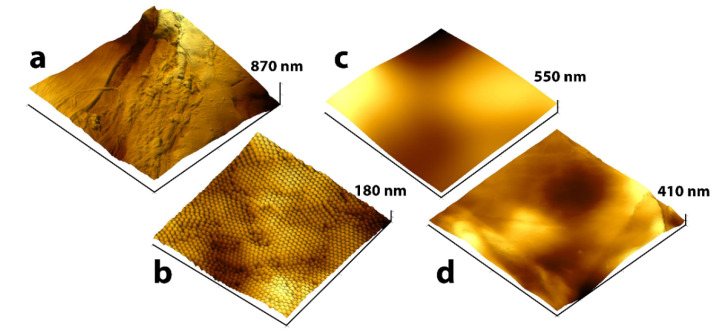
Three-dimensional AFM images of paper surface (**a**) before and (**b**–**d**) after treatment with pre-coating blends: (**b**) 8% PEVA emulsion, (**c**) 8% PVOH solution, and (**d**) 1% CC solution.

Atomic force microscopy (AFM) was used to further investigate the surface morphology and properties on a microscale. Figure 3a shows the irregularities and fibrous structure of the paper surface prior to coating. The roughened surfaces, in addition to the low adhesion of cellulose for silicone, is the primary reason why high quantities of silicone are required for an efficient coating. To improve the adhesion of silicone to the surface of the paper, the pre-coating layer should effectively “smoothen” the surface morphology, thereby reducing the roughness as much as possible. It was not possible to obtain AFM images of the paper coated with PS suspension due to the high mobility of polymer particles on the paper surface. In addition, particle mobility on the surface indicates the absence of chemical interactions between PS particles and cellulose, further reducing the potential of the PS blends as pre-coating layers. Figure 3b shows PEVA particles tightly packed on the paper surface. The layers of polymer particles (or thin film of crosslinked nanospheres) fully covered the natural fibrous morphology of the paper; however they did not eliminate the natural roughness of the paper surface completely. Figure 3c shows a different effect of the PVOH solution based pre-coating layer treatment compared to the previously examined coatings. After solvent evaporation, a uniform film of the polymer formed on the paper surface smoothing the fibrous morphology of the paper and reducing natural roughness. Figure 3d shows that the CC pre-coating blend did not fully smoothen the fibrous structure of the paper surface or significantly reduce its roughness. Moreover, this sample was quite easily damaged by the cantilever during the AFM study. The fragility of this pre-coating layer also confirmed the earlier conclusion that CC does not satisfy the requirements for pre-coating blends.

Table 1 shows the values of microscale surface roughness Sa and smoothness coefficient k. Smoothness coefficient is the ratio of the 3D surface area of the sample to the AFM scan area. The lower the coefficient value the smoother the surface. All pre-coating layers reduced paper roughness with the PVOH and PEVA samples producing the smallest smoothness coefficients. Based on the results of AFM analysis PVOH and PEVA pre-coating blends are the most suitable for release paper production.

### 3.3. Adhesive Tape Peel Test and Ink Drop Test

One of the most common tests used in the paper industry to determine the siliconization quality of a coating is the adhesive tape peel test. The simplest way to carry out this test is to press a piece of adhesive tape to the siliconized paper and after a period of time remove it from the paper with one sharp movement. If the adhesive tape gives a clean peel then the adhesion is categorized as low or very low, a key requirement for release paper.

**Figure 4:**
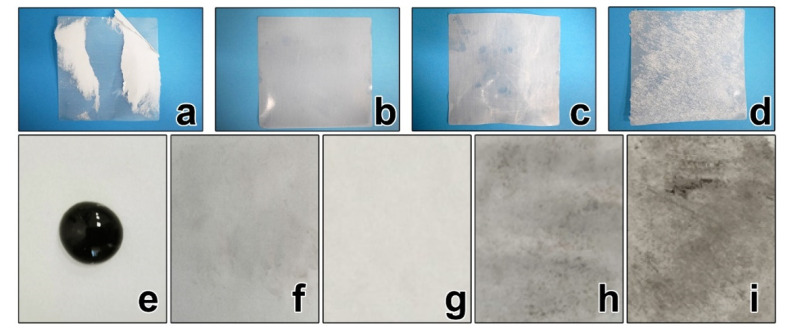
(**a**–**d**) Adhesive tape test. (**e**) Ink drop view and (**f**–**i**) ink drop test samples. Pre-coating layers: (**a,f**) PS suspension, (**b,g**) PEVA emulsion, (**c,h**) PVOH solution, and (**d,i**) CC solution.

Figure 4 shows adhesive tape test samples after 24 h adhered to siliconized release paper with each of the different pre-coating layers. Tape test samples were weighed prior to attachment to the paper and subsequent peeling. The mass difference of the tape samples for each pre-coating layer is shown in the Table 1.

Figure 4a shows the tape test sample peeled off the release paper prepared with PS suspension as the pre-coating blend. As can be seen from the image, considerable pieces of paper were present on the sticky side of the tape, suggesting that the siliconization of paper with the PS pre-coating layer occurred non-uniformly. SEM images of this type of pre-coating layer (Figure 2b) also confirmed that PS particles did not cover the entire surface of the paper.

Figure 4b shows the tape test for the sample prepared with the PEVA pre-coating layer. Results showed very little paper residue remaining adhered on the surface. It is therefore possible to conclude that when used as a pre-coating blend, PEVA enhances siliconization. 

Figure 4c shows the tape test sample prepared with the PVOH pre-coating layer. Despite some defects being visible on the adhesive layer, there were no paper residues, indicating that the siliconization occurred evenly over the entire surface. PVOH, however, contains hydrophilic OH^–^ groups, resulting in low adhesion to the hydrophobic silicone structure, which can also impact product quality. It can be seen from Figure 3c that PVOH did not create a specific surface topography and thus did not improve the adhesion of silicone to the surface. PVOH, however, remains a suitable choice for other types of paper with less roughness and correspondingly a higher price. 

Figure 4d shows samples prepared using the CC pre-coating blend. A uniformly distributed paper on the surface of the tape test sample could be seen due to the absence of siliconization. Moreover, it could be seen from SEM images that CC did not create a thin uniform coating on the paper surface, with which the ideal low anti-adhesion properties of release paper are associated. 

In addition, Table 1 shows that the mass of tape test sample after being peeled off from the siliconized paper treated with the PEVA pre-coating blend remained unchanged, further highlighting the anti-adhesive properties of this paper. The mass difference was also minimal in the case of the PVOH blend, but it was larger than the paper treated with PEVA. In the cases of the siliconized paper with pre-coating layers of the PS and CC, the weight of the tape test samples increased significantly, indicating the lack of release properties of the paper.

An “ink drop” test was also used to determine the quality of paper siliconization. A drop of ink was applied on the siliconized surface of release paper (Figure 4e) and then removed using filter paper or a napkin. The amount of ink remaining on the surface showed the quality of the release paper, whereas the less ink remaining, the more anti-adhesive properties the paper exhibited. 

Figure 4f shows that for the paper sample treated with PS suspension as the pre-coating blend, a significant quantity of ink pigment remained on the paper, highlighting poor paper siliconization.

Figure 4g shows the paper sample treated with the PEVA emulsion after ink drop removal from the paper surface. No pigment traces remained on the paper, further highlighting the anti-adhesion properties of this paper. This result was consistent with the results of the adhesive tape test, roughness measurements, and paper surface studies. The PEVA pre-coating layer completely covered the fibrous paper structure, resulting in reduced surface roughness. 

Figure 4h,i show residual pigment on the surfaces of the paper samples treated with PVOH solution and CC solution, suggesting that these paper samples do not have anti-adhesive properties. Even though PVOH effectively reduced the paper surface roughness, the results of the ink drop test and the adhesive tape test showed that the paper treated with PVOH did not exhibit the ideal anti-adhesive properties. CC had little effect in smoothing the natural structure of the paper as observed during SEM analysis and measurements of surface roughness. The results of the ink drop test and the adhesive tape test further confirmed that paper treated with CC solution did not exhibit anti-adhesive properties.

### 3.4. Raman Spectroscopy

**Figure 5:**
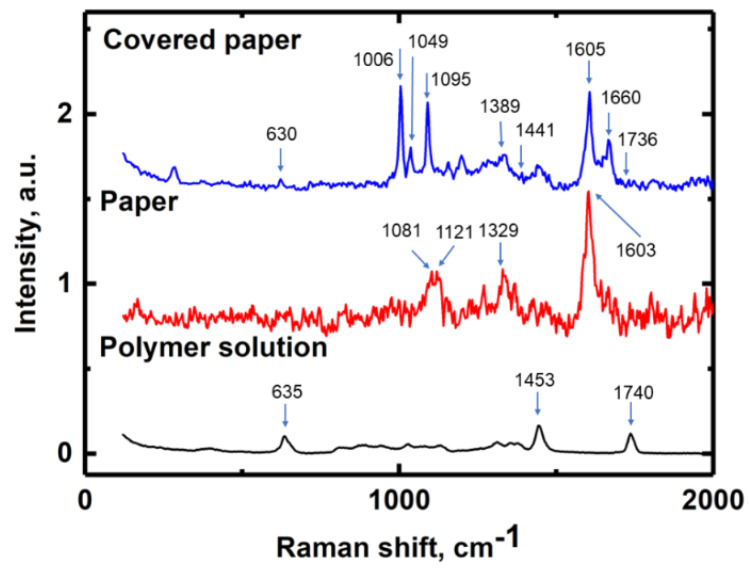
Raman spectra of paper base, PEVA (polymer) solution, and paper coated by PEVA. Arrows mark Raman shifts of main functional groups.

To further understand the interaction between the pre-coating layers and the paper base, the paper, polymers, and pre-coated samples were examined by Raman spectroscopy. Figure 5 shows the Raman spectra of the paper base, PEVA solution, and PEVA coated paper. Raman spectra for additional samples are presented in Supplementary Materials Figure S2. It is worth noting that Raman spectra of crosslinked and uncrosslinked PEVA were almost identical [24].

As high pulp paper was used in this study we should have been able to detect cellulose and lignin molecule traces on our spectra. Molecules of cellulose (Supplementary Materials Figure S3), lignin, and PEVA have many similar functional groups (C–C, C–H, C–O, OH, C–O–H, etc.) making analysis and identification challenging. Raman spectra of the paper base, PEVA solution and paper coated by PEVA are presented in Figure 5. To facilitate spectral interpretation, the attribution of the spectral lines is provided in Supplementary Materials Table S2 and in Supplementary Materials Figure S4. 

In the spectra of the individual PEVA polymer, the lines at 635 cm^–1^ and 1740 cm^–1^ could be attributed to a C=O deformation and stretching of the acetate moiety [25,26]. The stretch at 1453 cm^–1^ corresponded to the C–H deformation [25]. For the paper base spectra, lines 1081 cm^–1^ and 1121 cm^–1^ could be attributed to cellulous pyranose ring signal [27,28] and “breathing mode” of the pyranose ring [29]. Stretches at 1329 cm^–1^ and 1603 cm^–1^ could be attributed to lignin aromatic ring motion [25]. For the PEVA coated paper sample, stretches at 1006 cm^–1^ and 1095 cm^–1^ corresponded to cellulose [30], while stretches at 1049 cm^–1^ and 1605 cm^–1^ were lignin [30]. The stretch at 1660 cm^–1^ could be attributed to interactions between lignin and an acetate group [31,32]. The stretches at 1389 cm^–1^ and 1441 cm^–1^ corresponded to symmetric and asymmetric vibrations of the C–H bond present in the acetyl groups, respectively [31], whereas the stretch at 1736 cm^–1^ could be attributed to the vibration of the carbonyl group (C=O) [31]. The characteristic Raman lines for cellulose acetate could be observed at 1736 cm^–1^, 1435 cm^–1^, and 1382 cm^−1^, respectively [31]. It is clear there was an acetate group in the covered paper sample. The observed lines at 1736 cm^–1^, 1435 cm^–1^, and 1382 cm^−1^ suggested the occurrence of a chemical interaction between the acetate group of the PEVA blend and the cellulose of the paper base by way of esterification. 

Based on Raman data (Supplementary Materials Figure S5) obtained during confocal scanning, Raman signals at 1435 cm^–1^ and 1382 cm^−1^ were detected up to 3–4 um in depth from surface. The lignin and cellulose lines were observed at depths of up to 5 um. This suggests that the PEVA solution filled all pores on the paper surface, soaked into paper fibers, and then cured. The pre-coating layer and the paper base can interact in a number of ways. Depending on the surface tension and fluid properties the pre-coating layer will “flow” into the surface of the release paper essentially “fixing” on the surface. Secondly, there exists the possibility of chemical interactions between the pre-coating layer and the release paper itself, such as that between an acetate functional group and cellulose. The fixing properties of the PVOH solution were similar to those of PEVA; however, PVOH did not exhibit chemical interactions with the base paper. As such, PVOH did not bind as strongly to the paper base surface as did PEVA. 

### 3.5. Precoater Blends Comparison

Table 2 compares the properties of all four pre-coating blends. Parameters Sa and k were calculated from AFM measurements. Peel test, ink drop test, and uniformity were used to evaluate the quality of the pre-coating layers. The existence of chemical interactions between the PEVA pre-coating layer and the base paper was suggested from Raman experiment data. In each test, the PEVA pre-coating blend showed the most favorable properties as a pre-coating layer in release paper applications. 

PVOH showed acceptable results in surface roughness and smoothness tests but significantly poorer results during ink drop tests. Results showed that other blends had critical disadvantages also. PS performed the poorest in most tests, and CC did not fully meet the requirement during the peel and ink drop tests. In addition to favorable surface roughness and smoothness tests, PEVA was shown to self-assemble into uniform layers of nanospheres of approximately 100 nm in diameter. Self-assembly has previously been observed for copolymers of PEVA and polyethylene glycol [30,33]. Based on the comparison of each of the pre-coating blends we can say that PEVA is the best option as a pre-coater blend to create RP from cheaper OPD paper.

### 3.6. Cost Reduction Calculation

Associated costs of release paper production were provided by the industrial partner (Arkhangelsk’s Paper factory, Russia). There are a number of costs associated with the production of release paper including epoxysilicone blends (approx. 0.014 USD/m^2^), the coating process (approx. 0.04 USD/m^2^), and the paper base, typically 100% cellulose-based paper, “Glassine”, 60 m^2^/g (approx. 0.1 USD/m^2^). The high cost of producing release paper price is due the desired properties of 1) smooth surface, 2) dense material capable of undergoing super calendered stages, and 3) the requirement for a surface modifying layer (usually PVOH). A ready-to-use product based on “Glassine” silicone paper costs approximately 0.16 USD/m^2^ to produce. The method used in this work allowed us to use a cheaper paper (less than 0.04 USD) with the same density of 60 m^2^/g. Moreover, the intermediate PEVA layer costs 0.02 USD/m^2^ including reagents and application of the coating process. Whereas the consumption of epoxysilicone remains constant irrespective of the coating material, the use of PEVA with OPD paper is significantly more cost efficient than using PVOH and MG kraft paper. The minimum cost of this method is around 0.08 USD/m^2^ and could be made commercially available for around 0.11 USD/m^2^. 

## 4. Conclusions

This work investigates the effectiveness of four different polymeric solutions as pre-coating layers. The surface properties of paper treated with PS, PVEA, PVOH, and CC were investigated by SEM and AFM. The surface roughness values were recorded, and the ink drop test and adhesive tape peel-test were performed. It was found that the pre-coating blend based on 8% PEVA solution is the most suitable for release paper manufacturing. The improvement of the release paper properties by the pre-coating layer can be explained largely due to mechanical interaction of PEVA and the cellulose paper base. The use of PEVA as a pre-coating layer with OPD paper is environmentally friendly and has the capacity to lower the cost of release paper production without compromising final product quality. Significantly, PEVA pre-coating layers can be extended to cheaper pulp paper, allowing the production of high quality release paper from virtually any type of paper containing cellulose. This decrease in cost is critical for low margin markets and industries

## Figures and Tables

**Table 1:** Optimal active component concentrations for different types of pre-coating layer blends; adhesive tape peel test (ATPT) mass difference and surface morphology parameters for the paper samples with and without pre-coating layers. Sa—surface roughness, k—smoothness coefficient.

Sample	Optimal Active Component Concentration, %	ATPT Mass Difference, g	Sa, nm			k		
			Scan Size, μm					
			1	5	10	1	5	10
Paper	-	-	51	272	789	1.06	1.26	1.53
PS	3	0.69	-					
PEVA	8	0.01	2	9	261	1.01	1.02	1.10
PVOH	8	0.12	0	142	531	1.00	1.03	1.12
CC	1	0.50	10	97	129	1.06	1.14	1.20

**Table 2:** Pre-coating blends comparison.

Sample	Sa	k	Peel Test	Ink Drop Test	Link with Paper	Uniformity
PS	n/a	n/a	−	±	−	−
PEVA	+	+	+	+	±	+
PVOH	+	+	±	−	−	+
CC	+	−	−	−	−	±

## Supplementary Materials

The following are available online at https://www.mdpi.com/2079-4991/10/8/1436/s1, Figure S1: General scheme of commercial rotary printing press for offset printing, Table S1: The change of the mass of adhesive tape during the peel test, Figure S2: Raman spectra of the paper samples treated with pre-coating blends, Figure S3: The scheme of molecules: a) cellulose, b) PEVA, c) part of the lignin molecule, Figure S4: An attribution of the most part of Raman lines, Table S2: An attribution of the most part of Raman lines. Figure S5: Raman spectra into the depth of the sample. a) Optical image, Raman spectra in the different points; c–e) Raman cross sections into the depth of the samples by different peaks; f) Raman spectra examples at the different depth for 1435 cm^–1^ line.
