# Supplementary material for: An Investigative Study on the Effect of Pre-Coating Polymer Solutions on the Fabrication of Low Cost Anti-Adhesive Release Paper
Source: Nanomaterials (Basel). 2020 Jul 23;10(8):1436. doi: 10.3390/nano10081436 (PMC7466641; doi:10.3390/nano10081436)
Supplement: Supplementary file 1 [file nanomaterials-10-01436-s001.pdf]

nanomaterials

Article

An Investigative Study on the Effect of Pre-Coating Polymer Solutions on the Fabrication of Low Cost Anti-Adhesive Release Paper

Semen Vasilev [1,2], Andrey Vodyashkin [3,4], Daria Vasileva [2,5], Pavel Zelenovskiy [2], Dmitry Chezganov [2], Vladimir Yuzhakov [2], Vladimir Shur [2], Emmet O'Reilly [1] and Alexandr Vinogradov [3,*]

1 Department of Chemical Science, Bernal Institute, University of Limerick, V94 T9PX Limerick, Ireland; semen.vasilev@ul.ie (S.V.), emmet.oreilly@ul.ie (E.O'R.)
2 School of Natural Sciences and Mathematics, Ural Federal University, 620000 Ekaterinburg, Russia; daria.vasileva@ul.ie (D.V.); zelenovskiy@urfu.ru (P.Z.); chezganov.dmitry@urfu.ru (D.C); vladimir.juzhakov@urfu.ru (V.Y.); vladimir.shur@urfu.ru (V.Y.S)
3 ChemBio Cluster, ITMO University, 9 Lomonosova str., 191002 St. Petersburg, Russia; av.andrey2013@yandex.ru
4 Peoples' Friendship University of Russia (RUDN University), 6 Miklukho-Maklaya St., 117198 Moscow, Russia
5 Physical Department, University of Limerick, V94 T9PX Limerick, Ireland
* Correspondence: avv@scamt-itmo.ru;

Overview of the printing process; Silicone blends and poylmer pre-coaters were applied using a rotary printing press for offset printing. The plate cylinder was prepared so that the coatings cover it evenly and uniformly, and the offset cylinder to evenly transfer them to the paper. Water reservoir was filled with the polymer pre-coating blends.

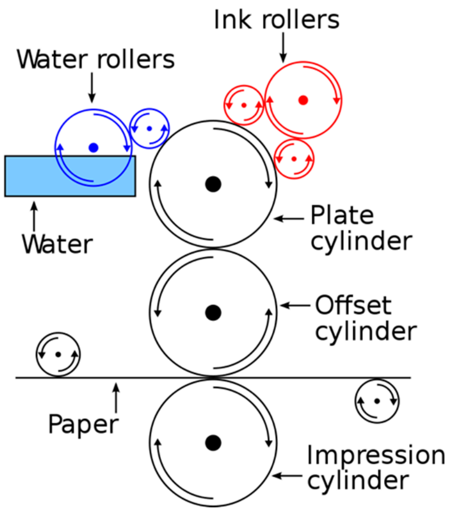

Figure S1. General scheme of commercial rotary printing press for offset printing.

Table S1. Change of mass of adhesive tape during Peel test.

	PS	PEVA	PVOH	CC
m_0	1.065	1.107	1.092	1.059
m_1	1.75	1.114	1.212	1.56
m_1-m_0	0.685	0.007	0.12	0.501

m_0 is the mass of adhesive tape before gluing to paper;
m_1 is the mass of adhesive tape after peeling off paper.

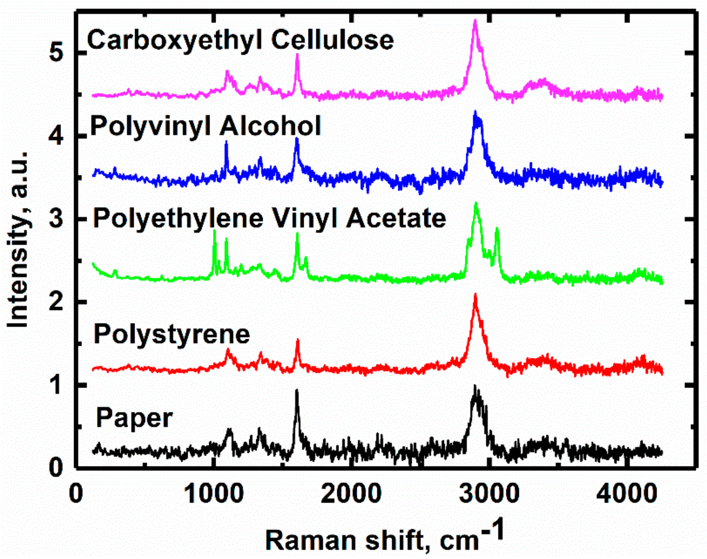

Figure S2. Raman spectra of the paper samples treated with a precoating blends.

Paper sample, 3% PS suspension, 8% PEVA emulsion, 8% PVOH solution, and 1% CC solution.

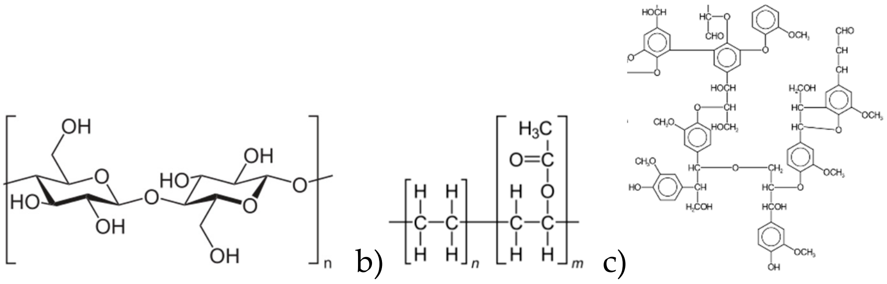

Figure S3. Chemical structure of a) cellulose, b) PEVA, c) lignin polymer.

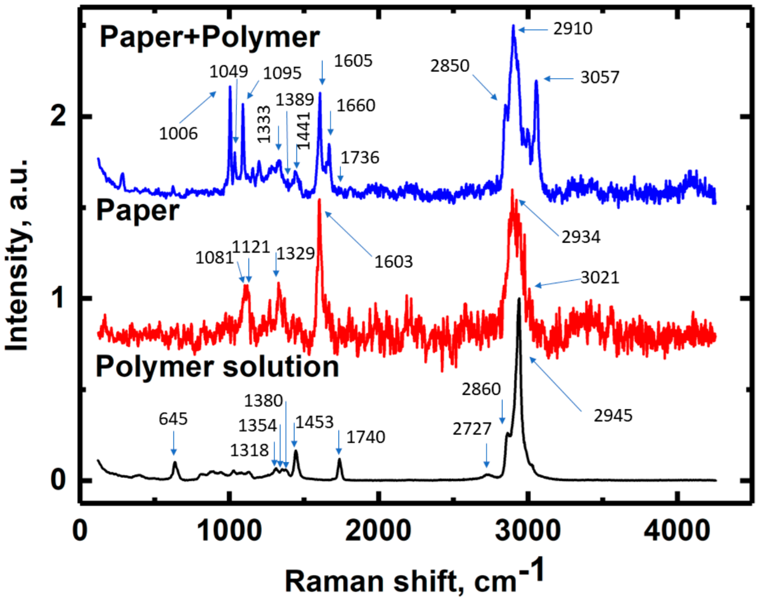

Figure S4. Raman Spectra identifying all significant Raman stretches and their respective frequencies.

Table S2. Respective frequencies of common Raman stretches.

Line	PEVA solution	Paper	Paper, covered by PEVA solution
635	C=O deformation [1]		C=O deformation [1]
1006			Cellulose [2]
1049			Lignin [2]
1081		Symmetric ring breathing mode of C-O-C [3] Cellulous pyranose ring signal [6]	
1095			Cellulose [2]
1121		Breathing mode of pyranose ring [3]	
1318	CH deformation vibrations		
1329		Lignin	
1333			Holocellulose [2,5] C–H deformation [1]
1354	C–H deformation [1]		
1380	C–H deformation [1]		
1389			symmetric vibrations of the C-H bond present in the acetyl groups [6]
1441			Asymmetric vibrations of the C-H bond present in the acetyl groups [6]
1453	C–H deformation [1]		
1605		Lignin (aromatic ring motion) [2]	
1603			Lignin (aromatic ring motion) [2]
1660			Lignin [2]
1736			vibration of the carbonyl group (C=O [6]
1740	vibration of the		

	carbonyl group (C=O [1]		
2727	Aliphatic C–H stretching [1]		
2850			Aliphatic C–H stretching [1]
2860	Aliphatic C–H stretching [1]		Aliphatic C–H stretching [1]
2910			Cellulose [2]
2934		C-H stretching cellulose [6]	
2945	Aliphatic C–H stretching [1]		
3021		Lignin [2]	
3057			Lignin [2]

Raman investigated the depth of paper covered by PEVA (Sample #2). Measurement was done at 3 different points (Line 1, 2 and 3 on the Figure S5b). The line intensity dramatically decreases at a depth of more than 3-4 um (Figure S5f).

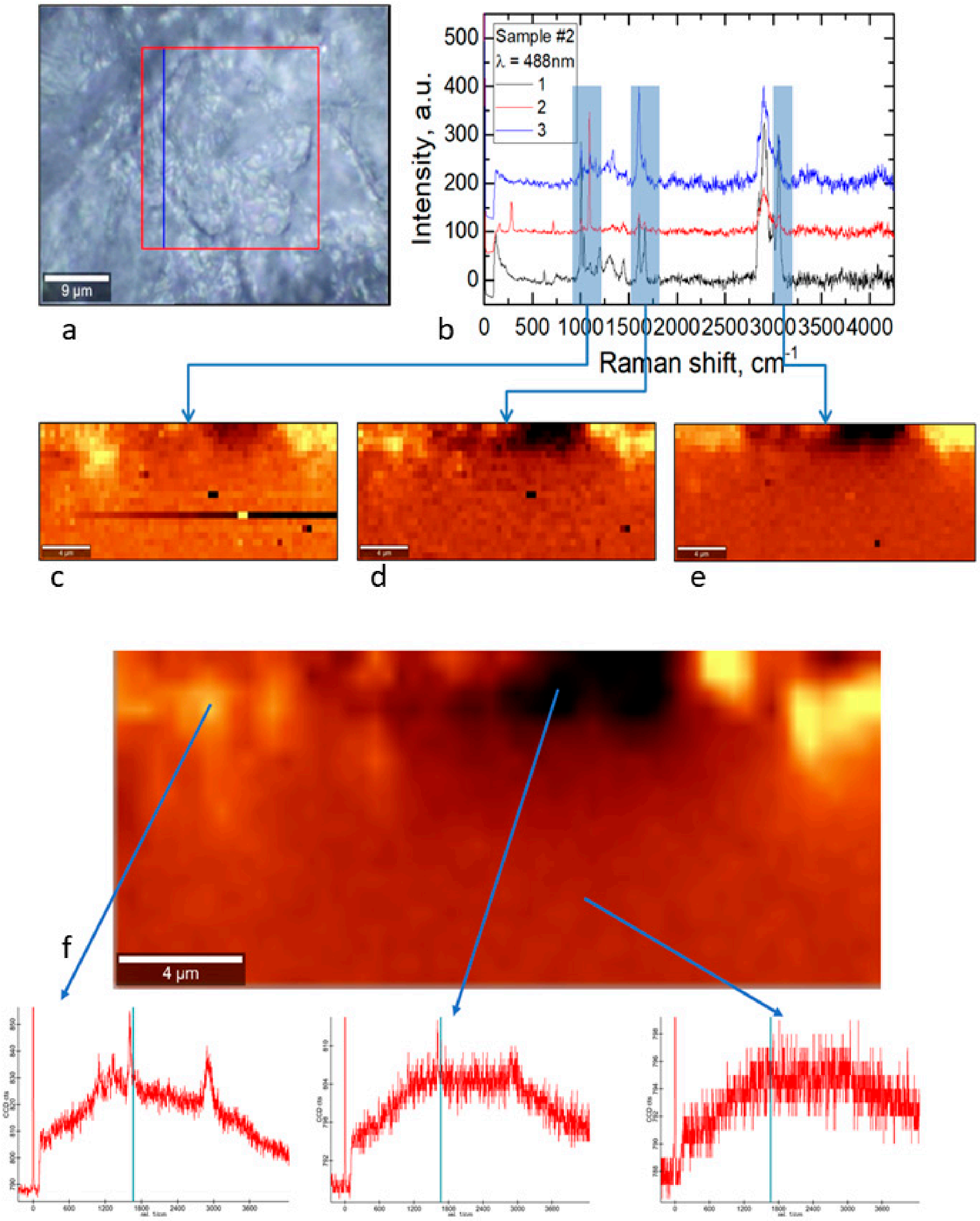

Figure S5. Raman spectra into the depth of the sample. a) Optical image, Raman spectra in the different points, c-e) Raman cross sections into the depth of the samples by different peaks, f) Raman spectra examples at the different depth for 1435 cm^{-1} line.

References

1. Sanoria, A.; Ulbricht, D.; Schuster, T.; Brüll, R. Monitoring crosslinking inhomogeneities in ethylene vinyl acetate photovoltaic encapsulants using Raman microscopy. *Rsc Adv.* **2015**, *5*, 93522–93529, doi:10.1039/C5RA18988H.
2. Petrou, M.; Edwards, H.G.M.; Janaway, R.C.; Thompson, G.B.; Wilson, A.S. Fourier-transform Raman spectroscopic study of a Neolithic waterlogged wood assemblage. *Anal. Bioanal. Chem.* **2009**, *395*, 2131–2138, doi:10.1007/s00216-009-3178-x.

3. Petrou, M.; Edwards, H.G.M.; Janaway, R.C.; Thompson, G.B.; Wilson, A.S. Fourier-transform Raman spectroscopic study of a Neolithic waterlogged wood assemblage. *Anal. Bioanal. Chem.* **2009**, *395*, 2131–2138, doi:10.1007/s00216-009-3178-x.
4. Chernev, B.S.; Hirschl, C.; Eder, G.C. Non-Destructive Determination of Ethylene Vinyl Acetate Cross-Linking in Photovoltaic (PV) Modules by Raman Spectroscopy. *Appl. Spectrosc.* **2013**, *67*, 1296–1301, doi:10.1366/13-07085.
5. Schenzel, K.; Fischer, S. NIR FT Raman spectroscopy - A rapid analytical tool for detecting the transformation of cellulose polymorphs. *Cellulose* **2001**, *8*, 49–57, doi:10.1023/A:1016616920539.
6. Sánchez-Márquez, J.A.; Fuentes-Ramírez, R.; Cano-Rodríguez, I.; Gamiño-Arroyo, Z.; Rubio-Rosas, E.; Kenny, J.M.; Rescignano, N. Membrane made of cellulose acetate with polyacrylic acid reinforced with carbon nanotubes and its applicability for chromium removal. *Int. J. Polym. Sci.* **2015**, *2015*, 1–12, doi:10.1155/2015/320631.
